# Pulmonary Vascular Permeability and Extravascular Lung Water Index in Patients with Liver Cirrhosis and Septic Shock

**DOI:** 10.3390/jcm13133796

**Published:** 2024-06-28

**Authors:** Kang-Wei Peng, Ming-Ling Chang, Rong-Nan Chien, Yung-Chang Chen, Ya-Chung Tian, Yun-Shing Peng, Hui-Chun Huang, Ji-Tseng Fang, Fa-Yauh Lee, Chih-Wei Yang, Ming-Hung Tsai

**Affiliations:** 1 Division of Gastroenterology and Hepatology, Chang Gung Memorial Hospital, Linkou Medical Center, Chang Gung University, Taoyuan City 333, Taiwan; forminpeng@gmail.com (K.-W.P.); mlchang8210@gmail.com (M.-L.C.);; 2 Division of Critical Care Nephrology, Kidney Institute, Chang Gung Memorial Hospital, Linkou Medical Center, Chang Gung University, Taoyuan 333, Taiwan; cyc2356@hotmail.com (Y.-C.C.); dryctian@yahoo.com (Y.-C.T.); cwyang@ms1.hinet.net (C.-W.Y.); 3 Department of Internal Medicine, Chang Gung Memorial Hospital, Chia-Yi 613, Taiwan; 4 Division of Gastroenterology and Hepatology, Department of Medicine, Taipei Veteran General Hospital, Faculty of Medicine, Yang-Ming University School of Medicine, Taipei 112, Taiwan; hchuang2@gmail.com (H.-C.H.);; 5 Division of General Medicine, Department of Medicine, Taipei Veteran General Hospital, Faculty of Medicine, Yang-Ming University School of Medicine, Taipei 112, Taiwan

**Keywords:** cirrhosis, sepsis, pulmonary vascular permeability index, extravascular lung water index

## Abstract

**Backgrounds and Aims**: Patients with cirrhosis are susceptible to sepsis and septic shock. Cirrhotic patients also have increased capillary permeability and are prone to developing volume overload. Patients with septic shock may have an enhanced pulmonary vascular permeability index (PVPI) and extravascular lung water index (EVLWI), both of which are associated with an unfavorable prognosis. It is plausible that pre-existing hyperpermeability may deteriorate when cirrhotic patients develop septic shock. However, it remains unknown whether PVPI and EVLWI can predict the prognosis of cirrhotic patients with septic shock. Pulse Indicator Continuous Cardiac Output (PiCCO) is an established tool to measure PVPI and EVLWI. Therefore, we conducted this retrospective study to investigate the prognostic significance of PVPI and EVLWI in cirrhotic patients with septic shock using PiCCO monitoring. **Methods**: We included 83 patients with liver cirrhosis and septic shock. EVLW indexed to actual body weight (aEVLWI), EVLW indexed to predicted body weight (pEVLWI), PVPI, disease severity scores, and other biomarkers were analyzed. We collected the PiCCO data on the first 2 days. **Results**: The overall 28-day mortality was 43.4%. The values of PVPI, aEVLWI, and pEVLWI on day 2 (PVPID2, aEVLWID2, EVLWID2) were significantly higher in non-survivors. The discriminating power of PVPID2 and EVLWID2 to predict 28-day mortality was tested using the area under a ROC curve. The areas under ROC curves (mean ± SEM) were 0.713 ± 0.061 and 0.650 ± 0.063 for PVPID2 and pEVLWID2. In the multivariate analysis, PVPID2, bilirubin, and lactate were independent factors which predicted 28-day mortality. **Conclusions**: Higher levels of PVPID2 and pEVLWID2 are associated with higher 28-day mortality rates in cirrhotic patients with septic shock. PVPI and pEVLWI may be useful to guide fluid management in this clinical setting.

## 1. Introduction

Septic shock is a common complication of liver cirrhosis [1]. Managing patients with septic shock involves balancing the need for adequate fluid resuscitation while avoiding complications associated with excessive fluid administration. Overloading the body with fluids can negatively impact prognosis. The accurate monitoring of hemodynamic indices in septic shock patients holds paramount importance in reducing mortality rates. Pulse Indicator Continuous Cardiac Output (PiCCO) monitoring is an innovative technique that has been used in critical care. PiCCO is a transpulmonary thermodilution device, which provides valuable insights into factors such as preload, afterload, extravascular lung water (EVLW), and pulmonary vascular permeability index (PVPI) [2,3,4].

EVLW indicates the amount of fluid which is accumulated in the interstitial and alveolar spaces. An increase in EVLW is the hallmark of acute lung injury (ALI) and acute respiratory distress syndrome (ARDS) [2]. EVLW is also elevated in septic shock and critically ill [3,4,5] patients. EVLW is traditionally indexed to actual body weight (aEVLWI). However, it has been shown that EVLW indexed to predicted body weight (pEVLWI) is a better marker to predict the prognosis of ALI/ARDS, compared to aEVLWI [5].

The pulmonary vascular permeability index (PVPI) is computed as the ratio between the EVLWI and the pulmonary blood volume [6,7]. Therefore, the PVPI is an indirect way to evaluate the integrity of alveolocapillary barrier. Several studies have highlighted the diagnostic and prognostic significance of PVPI in different clinical settings. It has been shown that the levels of PVPI are higher in patients with ALI/ARDS compared to those with hydrostatic pulmonary edema [8]. This distinction suggests that PVPI might serve as a valuable tool in differentiating between cardiogenic and non-cardiogenic types of pulmonary edema [9]. Furthermore, increased levels of EVLWI and PVPI have been identified as independent factors associated with 28-day mortality in patients with septic shock [7,9,10]. Taken together, these findings underscore the potential utility of the EVLWI and PVPI as prognostic indicators, aiding in the risk stratification and management of patients with septic shock.

In liver cirrhosis and portal hypertension, endothelial nitric oxide synthase (eNOS)-derived nitric oxide (NO) production is up-regulated in both the splanchnic and systemic circulation [11]. NO plays a pivotal role in arterial vasodilation, including the pulmonary vasculature [11]. Additionally, the eNOS-NO pathway is intricately involved in the regulation of vascular permeability mediated by Vascular Endothelial Growth Factor (VEGF) [12,13]. Indeed, pulmonary permeability has been shown to be increased in patients with liver cirrhosis [14]. Importantly, pulmonary complications are not uncommon in critically ill cirrhotic patients treated by albumin infusion [1], which certainly raises concerns about the interplay between volume load and pulmonary vascular permeability. The ideal goal of adequate volume resuscitation and the suitable tool to monitor response continue to be topics under debate. These issues are especially relevant in cirrhotic patients with septic shock, in whom pre-existing hyperpermeability in pulmonary vasculature may make this group of patients particularly vulnerable [15].

Considering the hemodynamic impairments inherent to liver cirrhosis and the potential risk incurred by volume resuscitation, measurements of PVPI and EVLWI may represent an opportunity to improve the outcomes in this particular subgroup of patients. Despite the widely accepted utility of PiCCO in critical care, there are no data regarding the prognostic significance of the EVLWI and PVPI in critically ill cirrhotic patients with septic shock.

We hypothesized that the EVLWI and PVPI could serve as prognostic markers for cirrhotic patients suffering from septic shock. Therefore, we conducted this retrospective study to investigate the prognostic roles of the PVPI and EVLWI.

## 2. Methods

### 2.1. Patient Information, Data Collection, and Definitions

This study is a retrospective analysis of prospective observational studies of critically ill cirrhotic patients admitted to the ICU. This study was conducted with approval from the institutional review board of Chang Gung Memorial Hospital (IRB202400261B0, issued on 5 March 2024), Taiwan, and in accordance with the Declaration of Helsinki of the World Medical Association. Patient consent was waived due to the retrospective nature of this study.

The study included cirrhotic patients with septic shock admitted to the specific Gastroenterology ICU in Chang Gung memorial hospital, Lin-Kou, Taiwan. Exclusion criteria included patients with heart failure and hypovolemic shock.

### 2.2. Patients

The diagnosis of cirrhosis in patients was made through abdominal CT scans, abdominal ultrasonography images, or liver biopsy. Sepsis, defined as the presence of signs of life-threatening organ dysfunction caused by a dysregulated host response to infection, was determined by an increase in Sequential Organ Failure Assessment (SOFA) score of 2 points or higher. Septic shock was characterized by the need for vasopressors to maintain a mean arterial pressure of 65 mm Hg or greater and a serum lactate level exceeding 2 mmol/L (>18 mg/dL) in the absence of hypovolemia [16]. All patients underwent resuscitation with a standardized treatment protocol for the management of septic shock [17]. Fluid resuscitation included colloids and crystalloid solutions and was aimed at maintaining a normal cardiac index (CI), intra-thoracic blood volume index (ITBVI), and EVLWI [18].

### 2.3. Laboratory Investigations

Hematological and biochemical studies, blood cultures, urine sediment, urine culture, and ascitic fluid neutrophil count and culture were routinely performed at inclusion.

### 2.4. Disease Severity Scores

Meanwhile, the severity of disease was graded by the Child–Pugh system and the Model for End-stage Liver Disease (MELD), APACHE-II score, and SOFA-score [19,20,21,22].

### 2.5. Measurements of and Hemodynamic Parameters

The PiCCO catheter system employs a single thermal indicator technique to determine parameters like EVLW, cardiac output (CO), and various volumetric parameters. Cardiac output and EVLW were acquired through the injection of 15 to 20 mL of iced (<6 °C) 0.9% saline solution into the central venous catheter. These measurements were obtained in triplicate, and the recorded values were calculated as the average of the three measurements. Iced saline was introduced into the central venous catheter, while the thermistor tip on the femoral artery catheter monitored the downstream temperature changes within the abdominal aorta. The cardiac output was subsequently calculated using the Stewart–Hamilton method based on thermodilution curves measured in the descending aorta. This method provides accuracy comparable to that of pulmonary artery thermodilution. Volumetric variables were derived using cardiac output, the mean transit time of the thermal indicator, and the decay time of the thermodilution curve, as previously described [5]. All volumetric and hemodynamic variables were indexed to body surface area, with the exception of EVLW. The PVPI was calculated as the EVLWI divided by pulmonary blood volume.

The EVLWI based on actual body weight (aEVLWI) was calculated by dividing the absolute EVLW by the patient’s actual body weight in kilograms, which was measured daily using the patient bed scale. The EVLWI based on predicted body weight (pEVLWI) was calculated by dividing the absolute EVLW by the predicted body weight, which was calculated using the Devine formula. Predicted body weight (in kilograms) was determined as follows: predicted body weight = 0.91[height (in centimeters) − 152.4] + 50 for men or +45.5 for women [5].

### 2.6. Statistical Analysis

Descriptive statistics are expressed as mean ± SD. All variables were tested for normal distribution using the Kolmogorov–Smirnov test. Student’s *t*-test was used to compare the means of continuous variables and the normal distribution data. Otherwise, the Mann–Whitney *U* test was used. Categorical data were tested using the Chi-square (χ^2^) test. The times of survival were analyzed by the Kaplan–Meier method and compared between groups with the log-rank test. Meanwhile, risk factors were assessed using univariate analysis and multivariate analysis by using logistic regression to obtain independent risk factors. Discrimination was tested using the area under a receiver operating characteristic (ROC) curve to assess the ability of PiCCO parameters to predict 28-day mortality. ROC analysis was also performed to calculate the cutoff values, sensitivity, specificity, overall correctness, and positive and negative predictive values. The best Youden index (sensitivity + specificity −1) was also used to determine the best cutoff point to predict 28-day mortality. All statistical tests were two-tailed, and the significance level was set at *p* = 0.05 or less. Data were analyzed using SPSS version 20 for Windows (IBM, Armonk, NY, USA).

## 3. Results

Table 1 shows the baseline characteristics and demographic data of the patients. The overall 28-day mortality rate was 43.4%. There were no significant differences between the 28-day non-survivors and survivors in terms of age and sex distribution; both groups were predominantly male.

The non-survivors had higher disease severity scores and lactate levels. The PVPI on day 1 and both the aEVLWI and pEVLWI on day 1 were not significantly different between survivors and non-survivors. The values of PVPI, aEVLWI, and pEVLWI on day 2 were significantly higher in non-survivors (Table 1).

**Figure 1:**
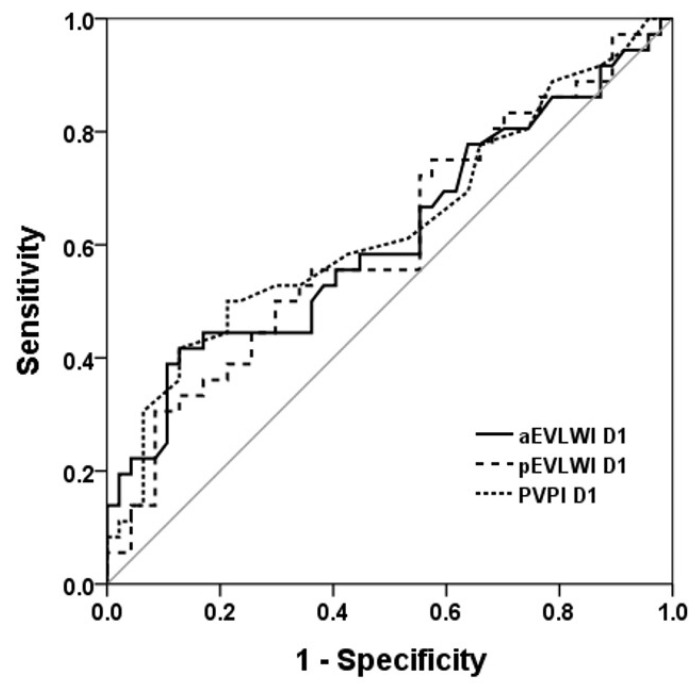
ROC curve to evaluate the discriminative capacity of aEVLWI, pEVLWI, and PVPI on day 1 to predict 28D outcome. ROC curve to evaluate the discriminative capacity of aEVLWI D1, p EVLWI D1, and PVPI D1 to predict 28-day outcome.

**Figure 2:**
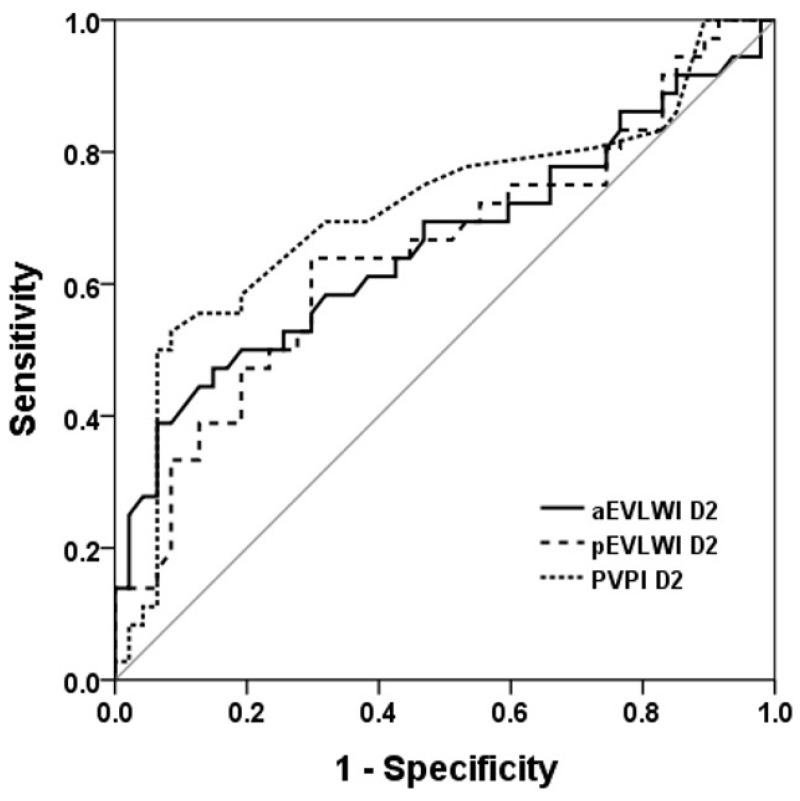
ROC curve to evaluate the discriminative capacity of aEVLWI, pEVLWI, and PVPI on day 2 to predict 28D outcome. ROC curve to evaluate the discriminative capacity of aEVLWI D2, pEVLWI D2, and PVPI D2 to predict 28-day outcome.

We applied receiver operating characteristic (ROC) curves to evaluate the discriminative capacity of aEVLWI, pEVLWI, and PVPI in distinguishing between survivors and non-survivors (Figure 1 and Figure 2). Of these parameters, PVPI D2 exhibited the most favorable AUROC (0.71 ± 0.061; 95% confidence interval, 0.593–0.832). The AUROC for pEVLWI D2 was 0.650 ± 0.063; 95% confidence interval, 0.527–0.772.

The cut-off values for PVPI D2 and pEVLWI D2 to predict 28-day mortality were obtained by analyzing the ROCs. Table 2 shows the predictive values of the chosen cutoff points (for PVPI D2, 2.65; for pEVLWI D2, 13.39 mL/Kg), which gave the best Youden index, for the prediction of 28-day mortality.

We further categorized patients into two groups based on their PVPI D2. Table 3 shows the demographic data and clinical characteristics of these two groups.

Table 4 shows the results of univariate and multivariate analyses to assess the associations between several variables and 28-day mortality. In the univariate analysis, lactate, bilirubin, INR, Child–Pugh, MELD, SOFA, APACHE II scores, aEVLWI D2, pEVLWI D2, and PVPI D2 were factors associated with 28-day mortality. In the multivariate analysis, we excluded those variables that were indeed different operationalizations of the same concepts. Two models were created. In model 1, we included lactate, bilirubin INR, and PVPI D2, while we substituted pEVLWI D2 for PVPI D2 in model 2. In model 1, lactate, bilirubin, and PVPI D2 were used as independent predictors to predict mortality, while lactate, bilirubin, and pEVLWI D2 were identified as independent predictors of mortality in model 2.

**Figure 3:**
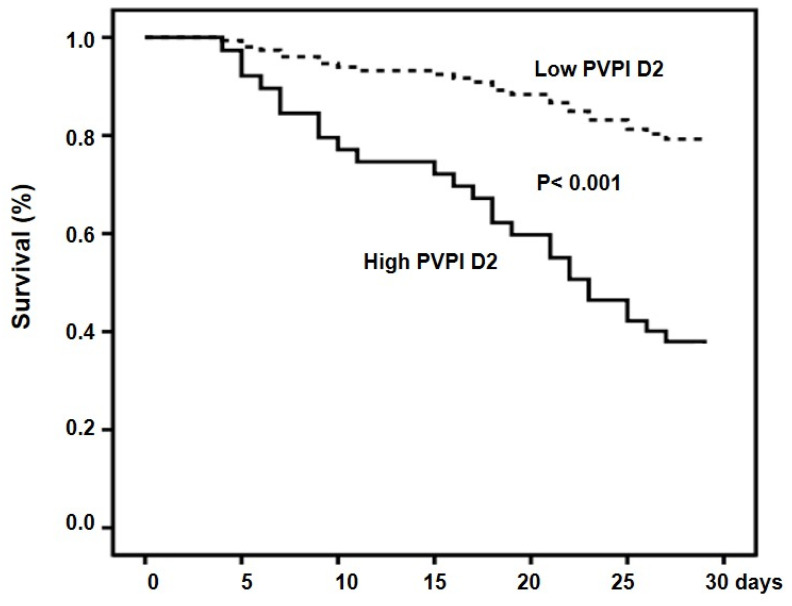
Cumulative survival in patients with high and low PVPI D2. Probability of survival was significantly lower in patients with high PVPI D2.

The cumulative rates of survival at 28 days were 71.7% and 17.4% for the low-PVPI D2 group and high-PVPI D2 group, respectively (*p* < 0.001) (Figure 3).

**Figure 4:**
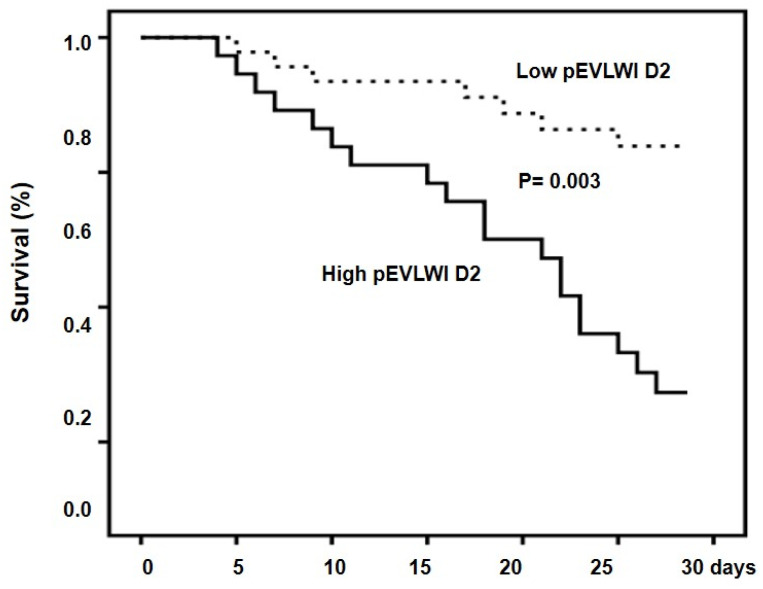
Cumulative survival in patients with high and low pEVLWI D2. Probability of survival was significantly lower in patients with high pEVLWI D2.

The cumulative rates of survival at 28 days were 71.7% and 37.8% for the low-EVLWI D2 group and high-PVPI D2 group, respectively (*p* = 0.003) (Figure 4).

**Figure 5:**
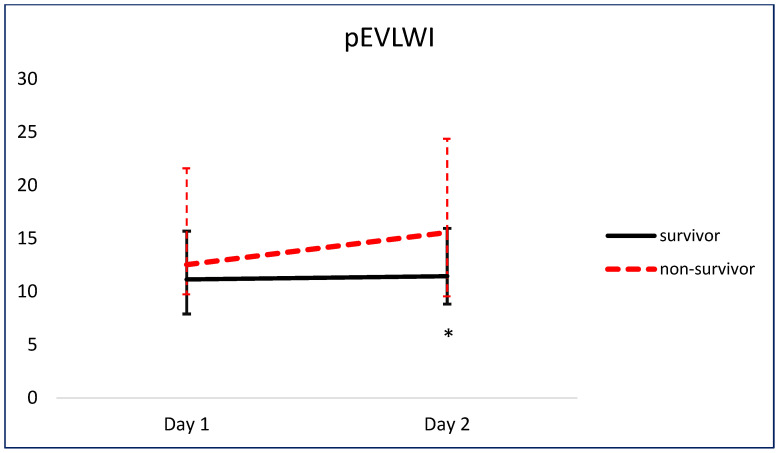
Comparison of serial pEVLWI between survivors and non-survivors. * *p* < 0.05.

**Figure 6:**
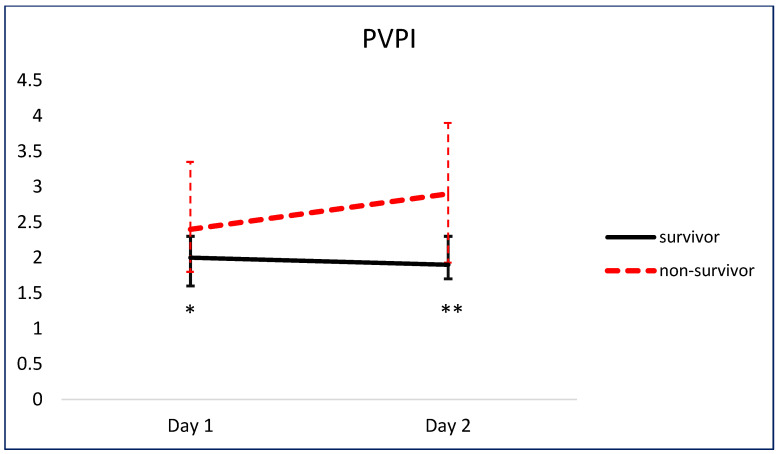
Comparison of serial PVPI between survivors and non-survivors. * *p* < 0.05. ** *p* < 0.01.

Figure 5 and Figure 6 show the comparison of serial pEVLWI and PVPI between survivors and non-survivors.

## 4. Discussion

The major findings of this study are as follows: (1) Higher levels of PVPI D2 and pEVLWI D2 are associated with 28-day mortality. (2) PVPI D2 is an independent factor to predict 28-day mortality in patients with liver cirrhosis and septic shock. The advanced hemodynamic assessment provided by PiCCO may aid in evaluating hemodynamic conditions, facilitating decision-making in critically ill patients. Although the PVPI is an indirect assessment of pulmonary permeability, it is the only method to evidence damage to the alveolo-capillary barrier and quantify the pulmonary leak at the bedside. Previous studies have often excluded cirrhotic patients when examining the associations between septic shock and these hemodynamic parameters. In this respect, our study represented the first to specifically examine the prognostic values of EVLWI and PVPI in cirrhotic patients with septic shock.

Among the cirrhotic patients admitted to hospital, one-third develop sepsis and 6% develop septic shock [15]. Vasodilatation is a hemodynamic hallmark of portal hypertension and liver cirrhosis. Once sepsis and septic shock ensue, this hemodynamic impairment deteriorates, further decreasing effective arterial volume and increasing the neurohumoral activity. In this context, timely fluid resuscitation is crucial for restoring intravascular volume and improving outcomes. However, positive fluid balance is an independent factor to predict mortality in critically ill patients with sepsis [23]. In this regard, cirrhotic patients may have even higher risk for developing pulmonary complications because of decreased oncotic pressure, impaired cardiac reserve [24], and pre-existing pulmonary hyper-permeability [14]. Recently, albumin infusion has been proposed to treat various complications of liver cirrhosis because of its oncotic and anti-inflammatory properties [25]. However, it may be detrimental in the presence of increased capillary permeability, leading to pulmonary edema and acute respiratory failure [25]. Maiwall et al. showed that albumin administration was associated with higher rates of shock reversal and lower rates of renal replacement treatment in cirrhotic patients with sepsis, when compared to plasamalyte infusion [26]. However, the hemodynamic effects could not be translated into survival benefits probably because of pulmonary complications. Lung edema occurred in 22% of patients in the albumin group. Similar safety concerns about lung edema were also observed in the ATTIRE [27] and CONFIRM [28] studies. Indeed, neural results in clinical trials may represent benefits for a certain subgroup and harm to others. In Maiwall’s study [26], patients with pneumonia and higher lactate levels and SOFA scores were at risk of pulmonary complications after albumin infusion. Interestingly, our results showed that high PVPI was associated with a higher SOFA score and higher rates of pneumonia (Table 3). Our findings may be pathophysiologically relevant. With respect to identifying the subgroup at risk of pulmonary complications, the EVLWI and PVPI may be utilized as criteria indicating the risk of fluid administration. High levels of PVPI indicate the presence of a pulmonary leak and caution against vigorous fluid administration, which is at a risk of increasing the EVLWI. High EVLWI values indicate that pulmonary edema is already present and interventions other than rapid volume expansion should be taken to improve hemodynamics. It is unknown whether PiCCO can help identify the subgroup of cirrhotic patients who can benefit from albumin infusion. It is also unknown if PiCCO can optimize the dose, duration, and frequency of albumin administration in critically ill patients with decompensated liver cirrhosis.

The relationship between EVLW and mortality has been explored at various time points. In a previous study, no significant differences were observed in the EVLWI and PVPI between survivors and non-survivors on day 1 following the onset of septic shock [6]. However, with the progression of sepsis, these variables exhibited significant intergroup differences by day 3 [6]. These findings are comparable with our results that the aEVLWI, pEVLWI, and PVPI were all significantly higher in non-survivors than in survivors on day 2. Interestingly, the PVPI was already significantly higher in non-survivors on day 1, suggesting increased vascular permeability precedes pulmonary edema in those patients who succumb and highlighting the potential ability of the PVPI to adjust the strategies of fluid administration in this clinical setting.

The mechanisms behind the pulmonary vascular leakage in sepsis are not fully understood. Recently, the roles of thrombin and alveolar epithelial-secreted protein isthmin1 have been explored [29,30]. Targeting thrombin or isthmin1 may represent a novel approach to treating pulmonary vascular leakage in sepsis.

This study has several limitations. Firstly, the modest sample size and the single-center nature of the study may restrict the generalizability of the results. Secondly, the retrospective design only permits the formulation of hypotheses regarding risk factors associated with mortality in cirrhotic patients with septic shock.

## 5. Conclusions

Higher levels of the PVPI and EVLWI are associated with increased mortality rates in patients with liver cirrhosis and septic shock. These hemodynamic parameters may serve as valuable indicators to guide fluid replacement strategies. Further studies are necessary to determine the potential benefits of EVLWI/PVPI-guided albumin infusion in this clinical setting.

## Figures and Tables

**Table 1:** Patients’ demographic data and clinical characteristics grouped according to 28-day mortality.

	All (*n* = 83)	28-Day Non-Survival (*n* = 36)	28-Day Survival (*n* = 47)	*p* Value
Age (years)	51 (46–63)	57 (43–63)	50 (47–64)	0.887
Sex (M/F)	72/11	31/5	41/6	0.881
CI D1 (L/min/m^2^)	4.49 ± 1.70	4.63 ± 1.53	4.39 ± 1.83	0.514
CI D2 (L/min/m^2^)	4.38 ± 1.73	4.61 ± 1.87	4.21 ± 1.60	0.300
ITBVI D1 (mL/m^2^)	949 ± 240	973 ± 245	931 ± 237	0.442
ITBVI D2 (mL/m^2^)	967 ± 260	965 ± 255	969 ± 266	0.933
Fluid balance D1 (mL)	1282 ± 1853	1622 ± 1623	1022 ± 1990	0.145
Fluid balance D2 (mL)	825 ± 1819	1420 ± 1901	368 ± 1630	0.008
Accumulated fluid balance (mL)	2107 ± 2987	3042 ± 2946	1390 ± 2845	0.012
aEVLWI D1 (mL/kg)	10.40 (8.00–15.80)	11.20 (8.50–18.78)	10.10 (7.80–13.90)	0.075
aEVLWI D2 (mL/kg)	11.80 (8.30–17.10)	14.90 (8.83–23.58)	10.40 (7.90–14.70)	0.013
ΔaEVLWI (mL/kg)	0.91 ± 4.60	1.31 ± 6.68	0.60 ± 1.87	0.492
pEVLWI D1 (mL/kg)	11.28 (8.83–18.26)	12.54 (9.75–21.60)	11.15 (7.54–15.69)	0.096
pEVLWI D2 (mL/kg)	12.55 (9.18–21.1)	15.56 (9.56–24.38)	11.45 (8.81–15.95)	0.020
ΔpEVLWI (mL/kg)	1.34 ± 5.07	1.91 ± 7.40	0.91 ± 1.93	0.374
PVPI D1	2.00 (1.70–2.80)	2.40 (1.80–3.35)	2.00 (1.60–2.30)	0.042
PVPI D2	2.20 (1.803.10)	2.90 (1.93–3.90)	1.90 (1.70–2.30)	0.001
ΔPVPI	0.20 (0.84)	0.36 ± 1.10	0.08 ± 0.44	0.117
Maximal NE dose (μg/min)	22.90 ± 18.71	28.36 ± 22.66	17.10 ± 10.96	0.013
Bilirubin D1 (mg/dL)	8.41 ± 8.76	11.47 ± 9.46	6.08 ± 7.48	0.005
Bilirubin D2 (mg/dL)	9.23 ± 8.42	13.24 ± 9.59	5.90 ± 5.51	<0.001
INR D1	2.17 ± 0.70	2.39 ± 0.74	2.00 ± 0.63	0.014
INR D2	2.16 ± 0.86	2.49 ± 1.04	1.91 ± 0.59	0.007
Albumin D1 (g/dL)	2.54 ± 0.53	2.50 ± 0.52	2.57 ± 0.54	0.549
Albumin D2 (g/dL)	2.73 ± 0.45	2.74 ± 0.54	2.72 ± 0.37	0.879
Child–Pugh score D1	11.25 ± 2.04	11.97 ± 1.93	10.63 ± 1.95	0.005
Child–Pugh score D2	11.11 ± 2.01	11.77 ± 1.86	10.60 ± 1.99	0.009
SOFA score D1	13.72 ± 3.19	15.48 ± 2.41	12.18 ± 3.00	<0.001
SOFA score D2	13.99 ± 3.38	15.89 ± 2.81	12.51 ± 3.06	<0.001
APACHE II D1	28.56 ± 6.61	30.58 ± 5.59	26.82 ± 6.99	0.016
APACHE II D2	27.53 ± 7.34	30.91 ± 6.76	24.89 ± 6.73	<0.001
MELD D1	29.41 ± 7.86	32.06 ± 7.28	27.11 ± 7.70	0.007
MELD D2	29.49 ± 7.90	33.11 ± 7.36	26.67 ± 7.17	<0.001
CRP D1 (mg/L)	73.13 (40.13–111.18)	73.805 (41.17–109.60)	66.85 (37.69–137.21)	0.915
CRP D2 (mg/L)	75.02 (41.52–118.445)	63.54 (40.07–113.43)	77.90 (42.64–143.44)	0.500
PCT D1 (ng/mL)	5.15 (1.55–17.60)	2.95 (1.20–12.59)	5.88 (1.94–21.35)	0.237
PCT D2 (ng/mL)	6.18 (1.93–18.77)	4.82 (1.66–8.58)	11.78 (3.15–19.88)	0.055
Leukocyte D1 (×10^9^/L)	14.88 ± 10.19	17.71 ± 12.98	12.54 ± 6.40	0.039
Leukocyte D2 (×10^9^/L)	15.02 ± 8.31	16.20 ± 9.07	14.09 ± 7.63	0.288
Lactate D1 (mg/dL)	38.70 (21.20–77.15)	60.70 (35.38–122.63)	22.25 (16.92–53.88)	<0.001
Lactate D2 (mg/dL)	31.15 (18.45–57.80)	41.65 (24.7–135.53)	24.95 (16.43–36.08)	0.001
Need for MV (%)	76/83 (91.6%)	34/36 (94.4%)	42/47 (89.4%)	0.693
Pneumonia (%)	57/83 (68.7%)	26/36 (72.2%)	31/47 (66%)	0.542
ARDS (%)	10/83 (12.0%)	9/36 (25.0%)	1/47 (2.1%)	0.002
Massive pleural effusion (%)	5/83 (6%)	5/36 (13.9%)	0/47 (0%)	0.013

Abbreviations: CI, cardiac index; D1, day 1; D2, day 2; ITBVI, intrathoracic blood volume index; EVLWI, extravascular lung water index; aEVLWI, EVLW indexed to actual body weight; pEVLWI, EVLW indexed to predicted body weight; PVPI, pulmonary vascular permeability index; NE, norepinephrine; SOFA, Sequential Organ Failure Assessment; APACHE II, acute physiology and chronic health evaluation II; MELD, Model for End-Stage Liver Disease; CRP, C-Reactive Protein; PCT, procalcitonin; MV, mechanical ventilation; ARDS, acute respiratory distress syndrome.

**Table 2:** PVPI D2 and pEVLWI D2 to predict 28-day mortality.

	Sensitivity	Specificity	PPV	NPV	Accuracy
PVPI D2	71.67%	82.61%	91.49%	52.78%	74.70%
pEVLWI D2	63.89%	70.21%	62.16%	71.74%	67.47%

Abbreviations: EVLWI, extravascular lung water index; PVPI, pulmonary vascular permeability index; pEVLWI, EVLW indexed to predicted body weight; PPV, positive predictive value; NPV, negative predictive value; D2, day 2.

**Table 3:** Patients’ demographic data and clinical characteristics grouped according to PVPI day 2.

	High PVPI D2 (>2.65) (*n* = 23)	Low PVPI D2 (<2.65) (*n* = 60)	*p* Value
Age (years)	54 (41–62)	51 (47–65)	0.756
Sex (M/F)	18/5	54/6	0.158
CI D2 (L/min/m^2^)	4.56 ± 2.11	4.31 ± 1.57	0.569
ITBVI D2 (mL/m^2^)	965 ± 258	968 ± 262	0.964
aEVLWI D2 (mL/kg)	22.60 (17.60–29.40)	9.90 (7.70–12.53)	<0.001
pEVLWI D2 (mL/kg)	24.24 (18.42–28.45)	10.845 (7.67–13.04)	<0.001
PVPI D2	3.9 (3.2–5.8)	1.85 (1.70–2.30)	<0.001
Accumulated fluid balance (mL)	2875 ± 2717	1812 ± 3055	0.148
Child–Pugh score D2	11.22 ± 1.783	11.07 ± 2.11	0.769
SOFA score D2	15.83 ± 2.33	13.25 ± 3.47	<0.001
APACHE II D2	31.61 ± 6.39	25.88 ± 7.10	0.001
MELD D2	29.7 ± 7.21	29.4 ± 8.21	0.882
CRP D2 (mg/L)	89.89 (44.46–151.66)	56.51 (31.50–105.34)	0.016
PCT D2 (ng/mL)	3.04 (1.22–5.82)	5.07 (1.19–10.40)	0.733
Leukocyte D2 (×10^9^/L)	13.79 ± 9.39	15.57 ± 7.82	0.405
Lactate D2 (mg/dL)	26.55 (21.8–96.48)	29.4 (15.65–75.05)	0.238
Need for MV (%)	23/23 (100%)	53/60 (88.3%)	0.182
Pneumonia (%)	21/23 (91.3%)	36/60 (60%)	0.007
ARDS (%)	10/23 (43.5%)	0/60 (0%)	<0.001
Massive pleural effusion (%)	1/23 (4.3%)	4/60 (6.7%)	1.000
28-day mortality (%)	19/23 (82.6%)	17/60 (28.3%)	<0.001

Abbreviations: CI, cardiac index; D2, day 2; ITBVI, intrathoracic blood volume index; EVLWI, extravascular lung water index; aEVLWI, EVLW indexed to actual body weight; pEVLWI, EVLW indexed to predicted body weight; PVPI, pulmonary vascular permeability index; SOFA, Sequential Organ Failure Assessment; APACHE II, acute physiology and chronic health evaluation II; MELD, Model for End-Stage Liver Disease; CRP, C-Reactive Protein; PCT, procalcitonin; MV, mechanical ventilation; ARDS, acute respiratory distress syndrome.

**Table 4:** Univariate and multivariate analysis to predict 28-day outcomes.

Parameter	Beta Coefficient	Standard Error	Odds Ratios (95%CI)	*p*
Univariate logistic regression				
Age (years)	−0.002	0.017	0.998 (0.965–1.031)	0.883
Sex (M/F)	0.097	0.651	1.102 (0.308–3.945)	0.881
MAP D2 (mmHg)	−0.019	0.015	0.981 (0.953–1.010)	0.191
Albumin D2 (g/dL)	0.036	0.484	1.036 (0.401–2.678)	0.941
Bilirubin D2 (mg/dL)	0.095	0.033	1.100 (1.032–1.173)	0.003
INR D2	1.221	0.412	3.390 (1.512–7.603)	0.003
Hepatic encephalopathy	0.616	0.476	1.852 (0.728–4.712)	0.196
Ascites	−0.598	0.585	0.550 (0.175–1.730)	0.307
Serum creatinine D2 (mg/dL)	0.100	0.109	1.105 (0.892–1.369)	0.359
Sodium D2 (mEq/L)	0.034	0.029	1.035 (0.977–1.096)	0.244
Potasium D2 (mEq/L)	0.450	0.254	1.568 (0.953–2.581)	0.077
aEVLWI D2 (mL/kg)	0.103	0.035	1.109 (1.034–1.188)	0.004
pEVLWI D2 (mL/kg)	0.078	0.030	1.081 (1.019–1.147)	0.009
PVPI D2	0.556	0.206	1.743 (1.163–2.612)	0.007
Child–Pugh score D2	0.311	0.123	1.365 (1.072–1.738)	0.012
APACHE II D2	0.146	0.044	1.157 (1.061–1.262)	0.001
MELD D2	0.120	0.035	1.127 (1.051–1.208)	0.001
CRP D2 (mg/L)	−0.003	0.003	0.997 (0.990–1.004)	0.344
PCT D2 (ng/mL)	−0.012	0.015	0.988 (0.960–1.018)	0.429
Lactate D2 (mg/dL)	0.018	0.007	1.018 (1.005–1.032)	0.006
Multivariate logistic regression (Model 1)				
PVPI D2	0.561	0.221	1.753 (1.136–2.704)	0.011
Bilirubin D2 (mg/dL)	0.136	0.051	1.146 (1.036–1.268)	0.008
Lactate D2 (mg/dL)	0.017	0.007	1.017 (1.004–1.030)	0.010
Multivariate logistic regression (Model 2)				
pEVLWI D2	0.093	0.037	1.098 (1.020–1.181)	0.012
Bilirubin D2 (mg/dL)	0.124	0.050	1.133 (1.027–1.249)	0.012
Lactate D2 (mg/dL)	0.019	0.007	1.019 (1.005–1.033)	0.007

Abbreviations: EVLWI, extravascular lung water index; PVPI, pulmonary vascular permeability index; aEVLWI, EVLW indexed to actual body weight; pEVLWI, EVLW indexed to predicted body weight; APACHE II, acute physiology and chronic health evaluation II; MELD, Model for End-Stage Liver Disease; MAP, mean arterial pressure; CRP, C-Reactive Protein; PCT, procalcitonin; D2, day 2.

## Data Availability

The datasets used and/or analyzed during the current study are available from the corresponding author on reasonable request.

## Abbreviations

EVLWI, extravascular lung water index; PVPI, pulmonary vascular permeability index; aEVLWI, EVLW indexed to actual body weight; pEVLWI, EVLW indexed to predicted body weight; CI, cardiac index; ITBVI, intrathoracic blood volume index; SOFA, Sequential Organ Failure Assessment; APACHE II, acute physiology and chronic health evaluation II; MELD, Model for End-Stage Liver Disease; CRP, C-Reactive Protein; PCT, procalcitonin; ARDS, acute respiratory distress syndrome.
